# Enhanced visible light photocatalytic performance of CdS sensitized TiO_2_ nanorod arrays decorated with Au nanoparticles as electron sinks

**DOI:** 10.1038/s41598-017-01124-5

**Published:** 2017-04-20

**Authors:** Xin Gao, Xiangxuan Liu, Zuoming Zhu, Ying Gao, Qingbo Wang, Fei Zhu, Zheng Xie

**Affiliations:** 1 High-Tech Institute of Xi’an, Xi’an, 710025 China; 2 High-Tech Institute of Beijing, Beijing, 100085 China; 3 State Key Laboratory of New Ceramics and Fine Processing, School of Materials Science and Engineering, Tsinghua University, Beijing, 100084 China

## Abstract

In this paper, we propose a nanostructure with Au nanoparticles (NPs), as electron sinks, located at the most outside layer of CdS sensitized TiO_2_ nanorod arrays (TiO_2_ NRAs/CdS/Au). By the introduction of Au NPs, TiO_2_ NRAs/CdS/Au performs higher visible light photocatalytic capacity in the degradation of unsymmetrical dimethylhydrazine wastewater than TiO_2_ NRAs/CdS. The optimal deposition time for Au NPs is 30 s. The visible light induced degradation ability of TiO_2_ NRAs/CdS/Au (30 s) is 1.4 times that of TiO_2_ NRAs/CdS. The cycling stability of TiO_2_ NRAs/CdS is greatly enchanced after Au NPs decoration, which can maintain 95.86% after three cycles. Photoluminescence spectra and photoelectrochemical measurements were carried out to reveal the underlying mechanism for the improved visible light photocatalytic capacity of TiO_2_ NRAs/CdS/Au. This work demonstrates a promising way for the rational design of metal-semiconductor photocatalysts used in decomposition reaction that can achieve high photocatalytic efficiency.

## Introduction

TiO_2_ nanostructures, as environmentally friendly materials, have been widely studied for the last several dozen years^1–5^. However, the wide band gap (>3.0 eV) of TiO_2_ restricts its utilization of the visible light in the solar spectrum^6–8^, and the low charge-mobility often leads to the high recombination rate of photogenerated electrons and holes in TiO_2_^9^. To overcome the barriers, combining TiO_2_ with a narrow band-gap semiconductor is of considerable interests for practical application at a low cost^10^. A typical example is the CdS-TiO_2_ composite, whose spectral response can be extended to the visible light region owing to the narrow energy band gap (2.4 eV) of CdS^11, 12^. In addition, the good band gap matching between TiO_2_ and CdS allows electrons generated from CdS to be transferred to TiO_2_, which could effectively accelerate the separation of electrons and holes^13, 14^. In particular, highly ordered one dimensional TiO_2_ nanorod arrays (TiO_2_ NRAs) sensitized with CdS nanoparticles (NPs) have better photoelectrochemical and photocatalytic capacity, because the ordered one dimensional nanostructure is more in favor of carriers separation^2, 15–18^.

Although the durability of CdS-TiO_2_ composite is often challenged, its excellent photocatalytic performance still attracts intensive studies. To improve the durability of CdS-TiO_2_, not at the expense of its photocatalytic capacity, several researches have been carried out. One of the effective methods is to strengthen the interaction between CdS and TiO_2_, which is closely related with their surface structures^19, 20^. However, it is relatively hard to precisely control the surface structures in preparation. Coating a thin layer made of stable materials on CdS, such as TiO_2_^13^ and ZnO^21^, to protect it from photocorrosion is also a good choice. The coating layer could not only prevent CdS to be corroded, but also favor carriers separation due to the bandgap matching between CdS and the coating layer. Therefore, Au enters our line of sight.

Au is very stable and highly resistant to oxidation^22^. When Au NPs contact with an semiconductor, it can usually help interfacial charge-transfer process^23^. Therefore, loading Au NPs on the surface of TiO_2_ NRAs covered by CdS NPs may be a useful way to improve its stability as well as the separation of carriers. Several investigations have been reported about nanocomposites containing Au, CdS and TiO_2_^24–28^. However, Au NPs either are put in the most inside as the nuclears, or placed in the middle of the structures as bridges for the carriers transfer in these literatures. There is still no nanostructure that loading Au NPs, as the most outside layer, on the surface of TiO_2_ NRAs covered by CdS NPs.

The high-energy unsymmetrical dimethylhydrazine (UDMH) is an excellent propellant primarily used in the space industry as well as the military theme^29^. However, the frequent use of UDMH in recent years makes a lot of UDMH wastewater. Animal experiments have proved that UDMH is carcinogenic, which poses a serious danger to the environment and human beings^30^. Traditional methods dealing with UDMH wastewater usually consume much energy and lead to second pollution by adding salts^31^. Therefore, UDMH was chosen as the model pollutant to test the effect of Au NPs on the photocatalytic performance of TiO_2_ NRAs/CdS under visible light irradiation. To understand the underlying reason for the improved photocatalytic capacity after Au NPs decoration, photoluminescence (PL) spectra and photoelectrochemical measurements were carried out. Finally, the possible mechanism of charge transfer and the photocatalytic process for TiO_2_ NRAs/CdS/Au was proposed.

## Results and Discussion

**Figure 1:**
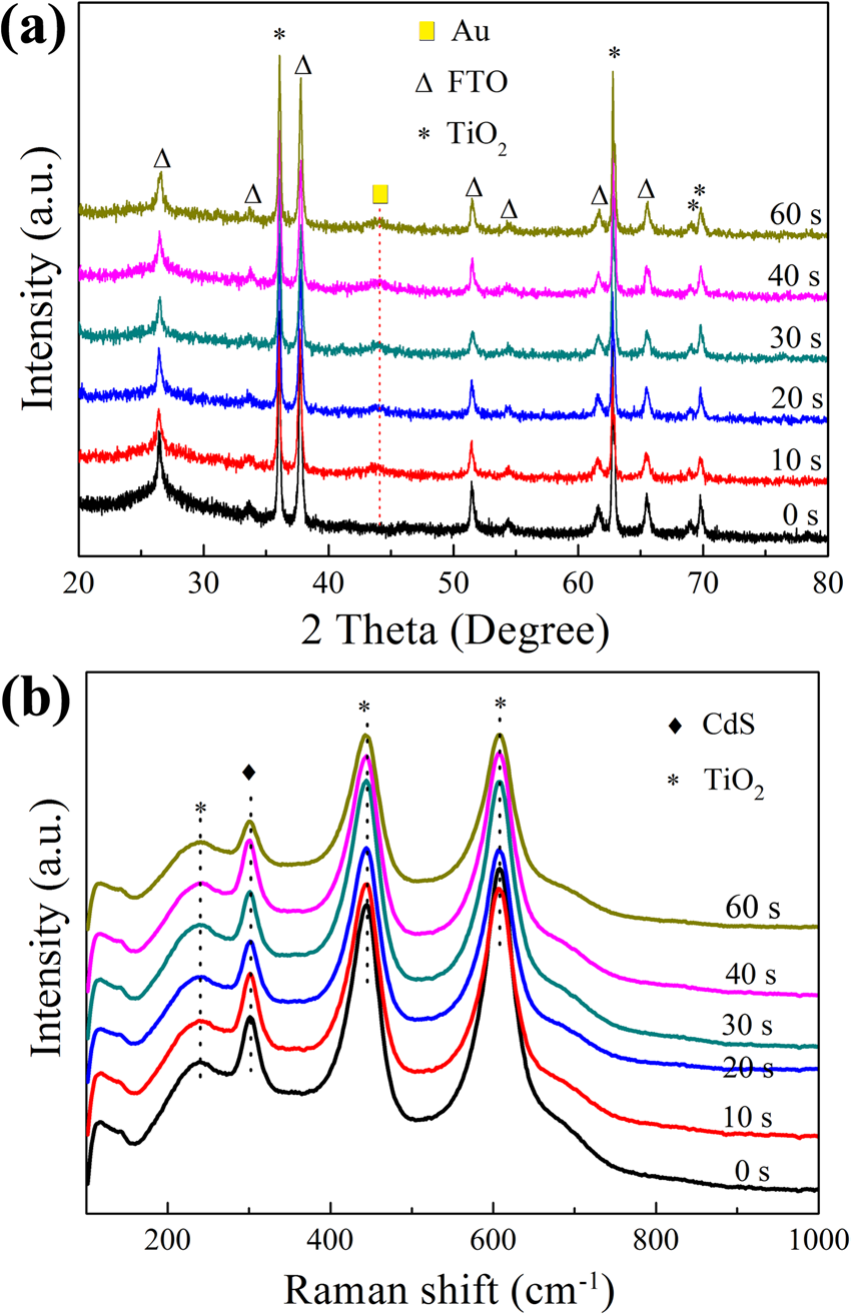
(**a**) XRD patterns and (**b**) Raman spectra of TiO_2_ NRAs/CdS/Au.

The XRD patterns of TiO_2_ NRAs/CdS decorated with different amounts of Au NPs are shown in Fig. 1(a). It can be observed that all the samples exhibit diffraction peaks centered at 2*θ* = 36.078°, 62.750°, 69.010° and 69.795°, indicating the presence of rutile TiO_2_ (PDF No. 21-1276). Characteristic peaks for CdS are not found in the XRD patterns, which may be due to the well dispersion and low concentration of CdS. After the deposition of Au NPs, a not very sharp peak at 2*θ* = 44.393° is present, belonging to Au NPs (PDF No. 04-0784). The diffraction peak of Au is very broad, which may indicate its small particle size. It is reported that when the grain size of the material is less than 10 nm, the diffraction peaks in the XRD patterns will be significantly broadened^32^. The particle size of Au NPs will be confirmed in the following TEM characterization. Other peaks marked by triangles in the XRD patterns are all from the transparent Fluorine-doped tin oxide glass (FTO) substrate (PDF No. 46-1088).

To further confirm the phase composition of the samples, Raman spectra were carried out as displayed in Fig. 1(b). The peaks at ~241.4 cm^−1^, ~445.6 cm^−1^ and ~609.5 cm^−1^ are three characteristic Raman active modes of rutile TiO_2_^33^, which is in accordance with the XRD patterns. The peak at ~302 cm^−1^ is resulted from the first order scattering of the longitudinal optical phonon mode generated by CdS^34, 35^, thus it can be proved that CdS is one of the components in the as-prepared samples. The peak at 117 cm^−1^ is caused by plasma emission of the Ar^+^ laser in the characterization^36^.

**Figure 2:**
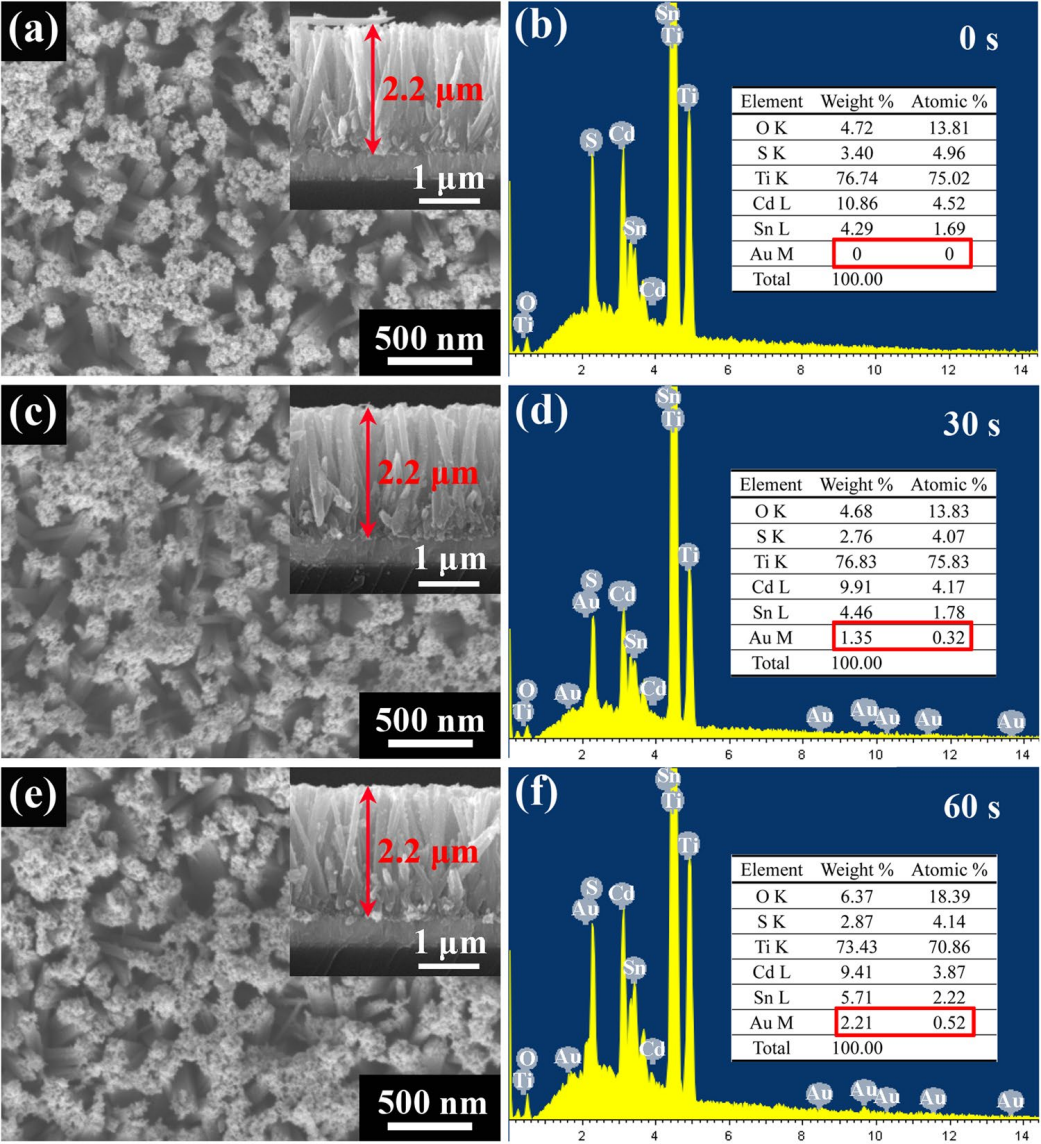
SEM and EDS images of TiO_2_ NRAs/CdS/Au samples: (**a** and **b**) 0 s, (**c** and **d**) 30 s, (**e** and **f**) 60 s.

SEM images of the as-prepared samples are shown in Fig. 2. The morphology of the bare TiO_2_ NRAs has been characterized by SEM in our previous work^37^. TiO_2_ nanorods uniformly grow on the FTO substrate and the typical nanorod is 2.2 *μ*m in length and 60 nm to 120 nm in diameter. After 15 cycles of CdS deposition, big CdS NPs are accumulated on the top of the TiO_2_ NRAs as shown in Fig. 2(a). By comparing the SEM images in Fig. 2(a,c and e), there is no recognizable change in the surface morphology of the TiO_2_ NRAs/CdS before and after Au NPs decoration, which may suggest the small particle size and low content of Au NPs. It is hard to discern the CdS distribution along the nanorod from the SEM images, which will be further clarified in the following TEM characterization. The nanorod length in all samples is *ca*. 2.2 *μ*m according to the cross-section images inset, corresponding with that of the bare TiO_2_ NRAs as reported in our previous work^37^. To reveal the different amounts of Au NPs among the samples, energy dispersive X-ray spectrum (EDS) analysis was carried out for the areas corresponding to Fig. 2(a,c and e). From the EDS result, it can be observed that the Au weight ratio is from 0 to ~2.21 when the deposition time increased from 0 s to 60 s, which indicates that more Au NPs were loaded on TiO_2_ NRAs/CdS. However, the content of Au NPs is still very low even in the TiO_2_ NRAs/CdS/Au (60 s) sample.

**Figure 3:**
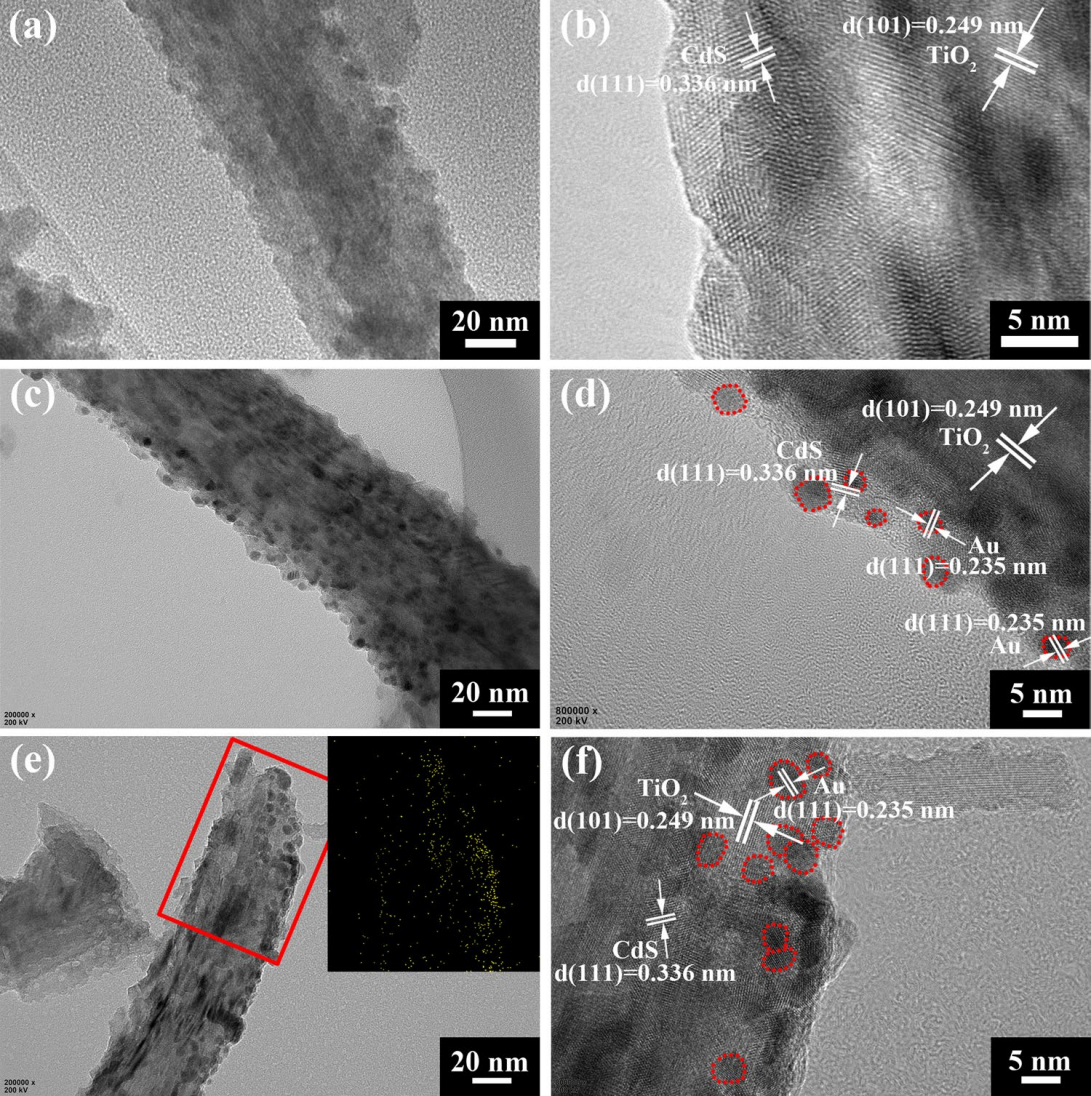
TEM images of TiO_2_ NRAs/CdS/Au: (**a** and **b**) 0 s, (**c** and **d**) 30 s, (**e** and **f**) 60 s. The inset of (**e**) is the distribution of Au NPs corresponding to the region marked by the red rectangle. Au NPs are marked by red circles in (**d** and **f**).

To further clarify the microstructure of TiO_2_ NRAs/CdS/Au, TEM characterization was carried out. Figure 3(a and b) show the morphology of the binary TiO_2_ NRAs/CdS. It can be seen that the TiO_2_ nanorod is covered with a thin layer made of CdS NPs. After ion sputtering Au NPs for 30 s, small Au NPs are dispersed in the relatively large CdS nanocrystals in Fig. 3(c and d). The high resolution TEM in Fig. 3(d) exhibits that the diameters of Au NPs are no more than 5 nm, and a considerable portion are even less than 3 nm. Increasing the ion sputtering time to 60 s, Au NPs grow bigger according to Fig. 3(e and f). Most grow to about 5 nm in diameter, causing some agglomerations. The size distributions of Au NPs in TiO_2_ NRAs/CdS/Au (30 s) and TiO_2_ NRAs/CdS/Au (60 s) were counted as shown in Fig. S1. The average particle size for TiO_2_ NRAs/CdS/Au (30 s) and TiO_2_ NRAs/CdS/Au (60 s) is 3.41 nm and 4.60 nm, respectively, indicating that Au NPs grow bigger with increase in ion sputtering time. The inserted picture in Fig. 3(e) is the TEM mapping of Au NPs corresponding to the region marked by the red rectangle. It can be seen that Au NPs are not uniformly dispersed in the sample, because they were deposited from the top to the bottom along the nanorod. The distributions of Ti, O, Cd and S in the specific portion selected for the mapping study are displayed in Fig. S2, which are all distributed evenly.

**Figure 4:**
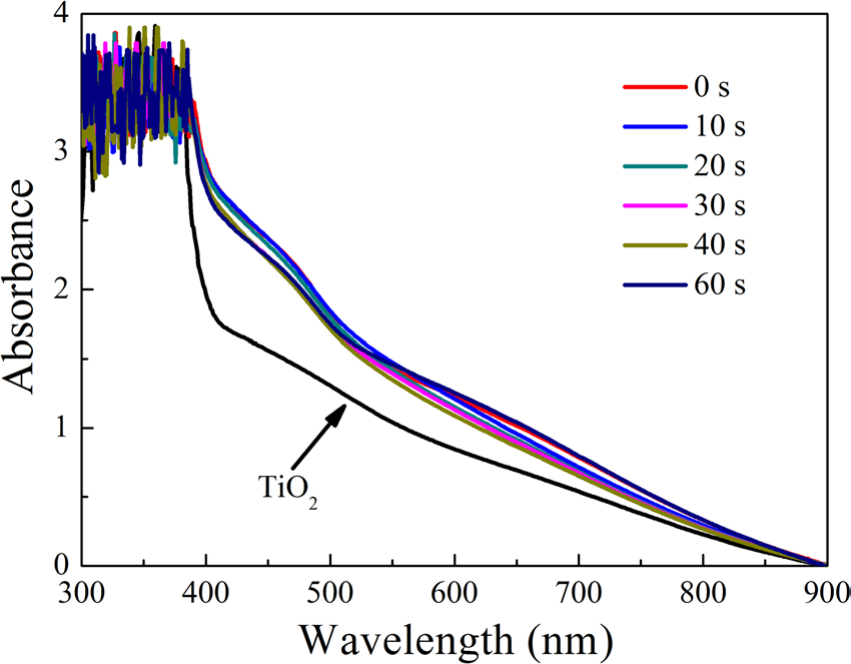
UV-vis absorption spectra of TiO_2_ NRAs/CdS/Au samples.

UV-vis spectra of the as-prepared samples are shown in Fig. 4. There is no obvious change in the visible light absorption of TiO_2_ NRAs/CdS after Au NPs decoration. The tiny differences in the absorption curves should attribute to the differences of the FTO substrates. It is strange that no plasma resonance absorption occurred after Au NPs decoration, which may be for the small particle size and the low distribution density of Au NPs as shown in the TEM images. Similar to the result in our experiment, it is reported that Au NPs with the diameter below 3 nm in toluene did not exhibit plasma resonance absorption, either^38^. In order to exclude the plasma resonance absorption from Au to be overwhelmed by the strong adsorption of CdS layer, we recorded the UV-vis absorption of Au NPs decorated TiO_2_ NRAs. As shown in Fig. S3, without the interference of CdS, TiO_2_ NRAs/Au (30 s) and TiO_2_ NRAs/Au (60 s) displayed no plasma resonance absorption, either, which demonstrated that Au NPs loaded on the surface of TiO_2_ NRAs/CdS truly did not cause the plasma resonance effect in our experiment.

**Figure 5:**
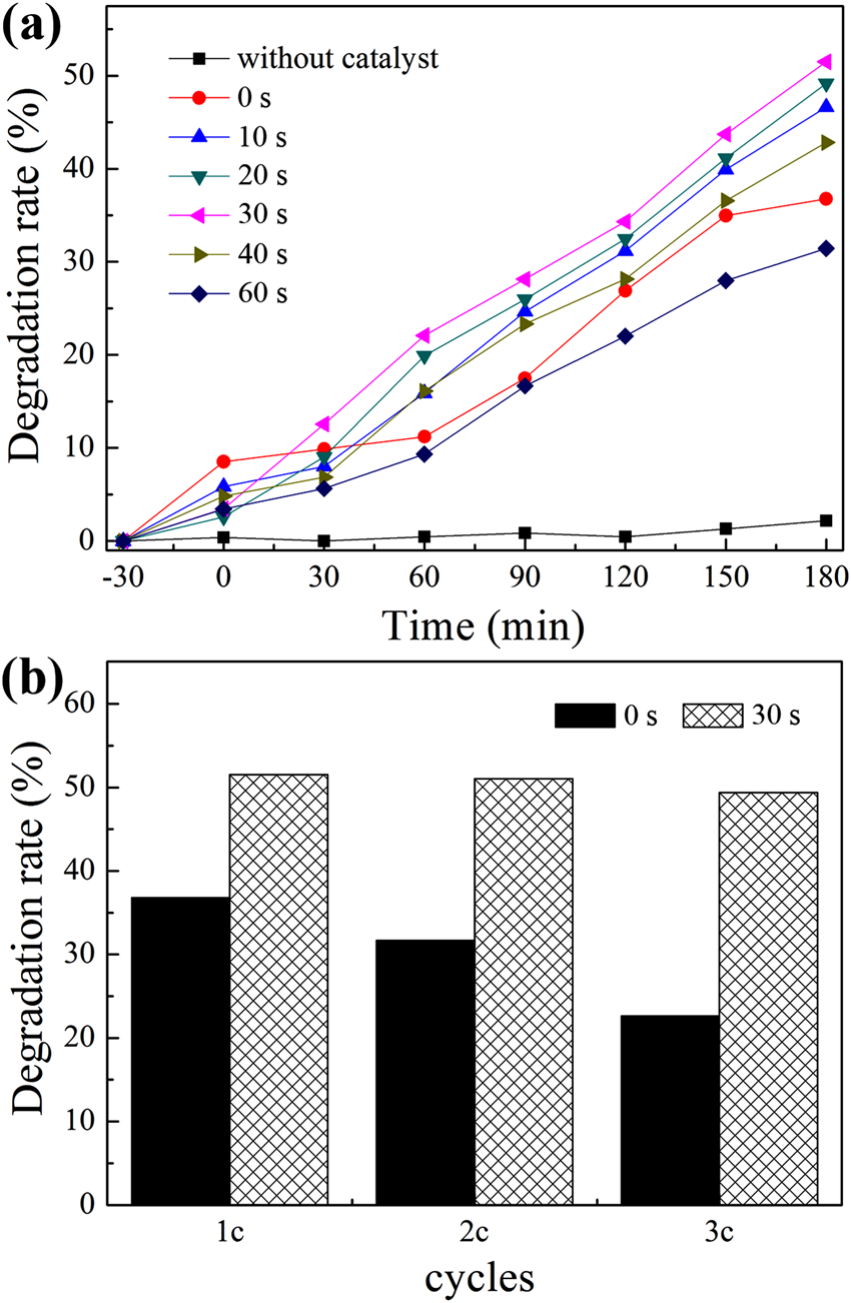
(**a**) Degradation curves of UDMH by TiO_2_ NRAs/CdS/Au under visible light irradiation, (**b**) The visible light photocatalytic durability of TiO_2_ NRAs/CdS and TiO_2_ NRAs/CdS/Au.

Figure 5(a) shows the photocatalytic performance of TiO_2_ NRAs/CdS/Au evaluated by the degradation of UDMH wastewater under visible light irradiation. As a comparison, UDMH degradations were also carried out without the addition of any photocatalyst and in the existence of TiO_2_ NRAs/CdS. Visible light irradiation for 180 min can only get about 2% degradation of UDMH, while the photodegradation rate of UDMH by TiO_2_ NRAs/CdS reaches 36.77% under the same condition. When employing TiO_2_ NRAs/CdS/Au as the photocatalyst, apparent enhancement of the photodegradation rates can be observed. By increasing the deposition time of Au NPs from 0 s to 30 s, the photodegradation rate of UDMH is improved moderately from 36.77% to 51.51%. And a 0.4 time increase was achieved by TiO_2_ NRAs/CdS/Au (30 s) compared with TiO_2_ NRAs/CdS. Au NPs, as electron sinks, can effectively retard the recombination of photogenerated electron-hole pairs by extracting electrons from CdS. Moreover, the small Au NPs provides huge specific surface area, therefore, electrons have a bigger chance to be trapped by dissolved oxygen and more ·O_2_^−^ with strong oxidizing property will be produced. The synergy effects contribute to the improved photocatalytic performance of TiO_2_ NRAs/CdS/Au (≤30 s). However, the photocatalytic performance deteriorates when continually increasing the deposition time (≥40 s). TiO_2_ NRAs/CdS/Au (60 s) can only obtain 31.52% degradation of UDMH. When more Au NPs are loaded (≥40 s), it reduces the active surface area in CdS, therefore, the generation of photoelectrons and holes in CdS and the UDMH molecular absorption will be hindered. In addition, the agglomeration of Au NPs aggravates with the increase of the deposition time, acting as recombination centers for carriers^39–41^. As a result, the photocatalytic property declines when overloading of Au NPs (≥40 s). In summary, it is of importance to optimize the amount of Au NPs to obtain an ideal photocatalytic capacity.

The cycling durability of the samples before and after Au NPs decoration was carried out under visible light irradiation and each cycle was conducted for 180 min. As shown in Fig. 5(b), the photodegradation rate of UDMH over TiO_2_ NRAs/CdS is found to be about 36.77% (once), 31.69% (twice) and 22.63% (third times), respectively, while it is 51.51% (once), 51.03% (twice) and 49.38% (third times) over TiO_2_ NRAs/CdS/Au (30 s). One can see that the introduction of Au NPs can significantly enhance the photocatalytic stability of TiO_2_ NRAs/CdS. The cycling stability of the ternary TiO_2_ NRAs/CdS/Au can maintain 95.86% after three cycles, much higher than 61.54% of the binary TiO_2_ NRAs/CdS.

To clarify the underlying mechanism for the enhanced photocatalytic capacity of TiO_2_ NRAs/CdS/Au, PL spectra, photocurrent density versus potential (*I-V*) and photocurrent density versus time (*I-T*) curves were measured.

**Figure 6:**
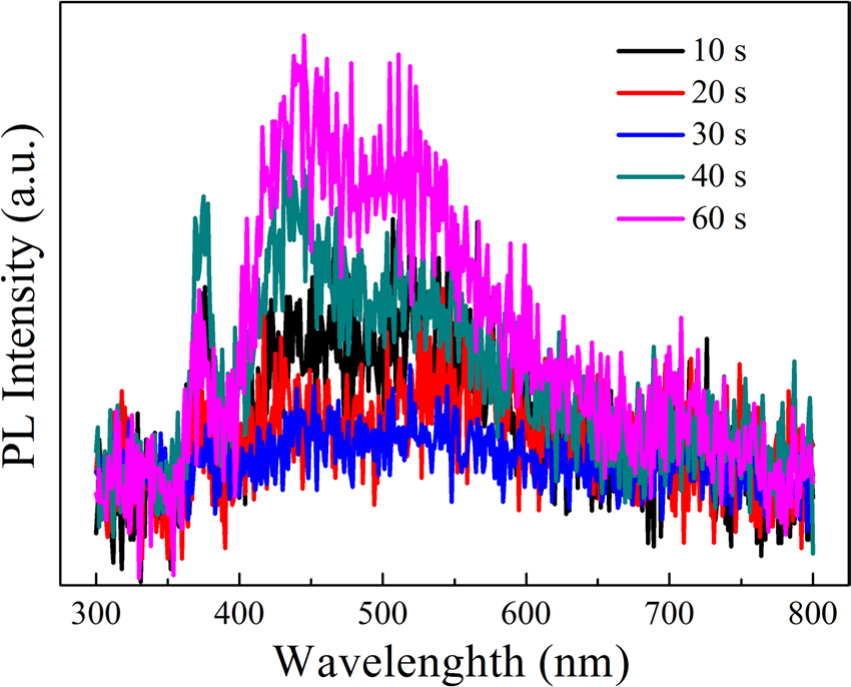
PL spectra of TiO_2_ NRAs/CdS/Au.

PL spectra of the TiO_2_ NRAs/CdS/Au are shown in Fig. 6. The peak located at ~425 nm can be ascribed to the self-trapped excitons in TiO_2_^42^. The other one at ~530 nm can be corresponding to the defects forming at the surface of TiO_2_ NRAs/CdS/Au^42–44^. PL intensity can reflect the separation efficiency of carriers^45^. Stronger intensity in PL peaks indicates higher recombination rates of carriers^46^. As we expected, the PL intensity for TiO_2_ NRAs/CdS/Au decays gradually with the increase in the deposition time (≤30 s), indicating more effective separation of photogenerated hole–electron pairs, thus contributing the enhanced photocatalytic activity for TiO_2_ NRAs/CdS/Au (≤30 s). When longer deposition time is conducted (≥40 s), the PL intensity for TiO_2_ NRAs/CdS/Au becomes stronger, suggesting that the separation of photogenerated electrons and holes get worse due to the agglomerated Au NPs as recombination centers. Therefore, the photocatalytic degradation rate began to decrease when the deposition time for Au NPs was more than 30 s.

**Figure 7:**
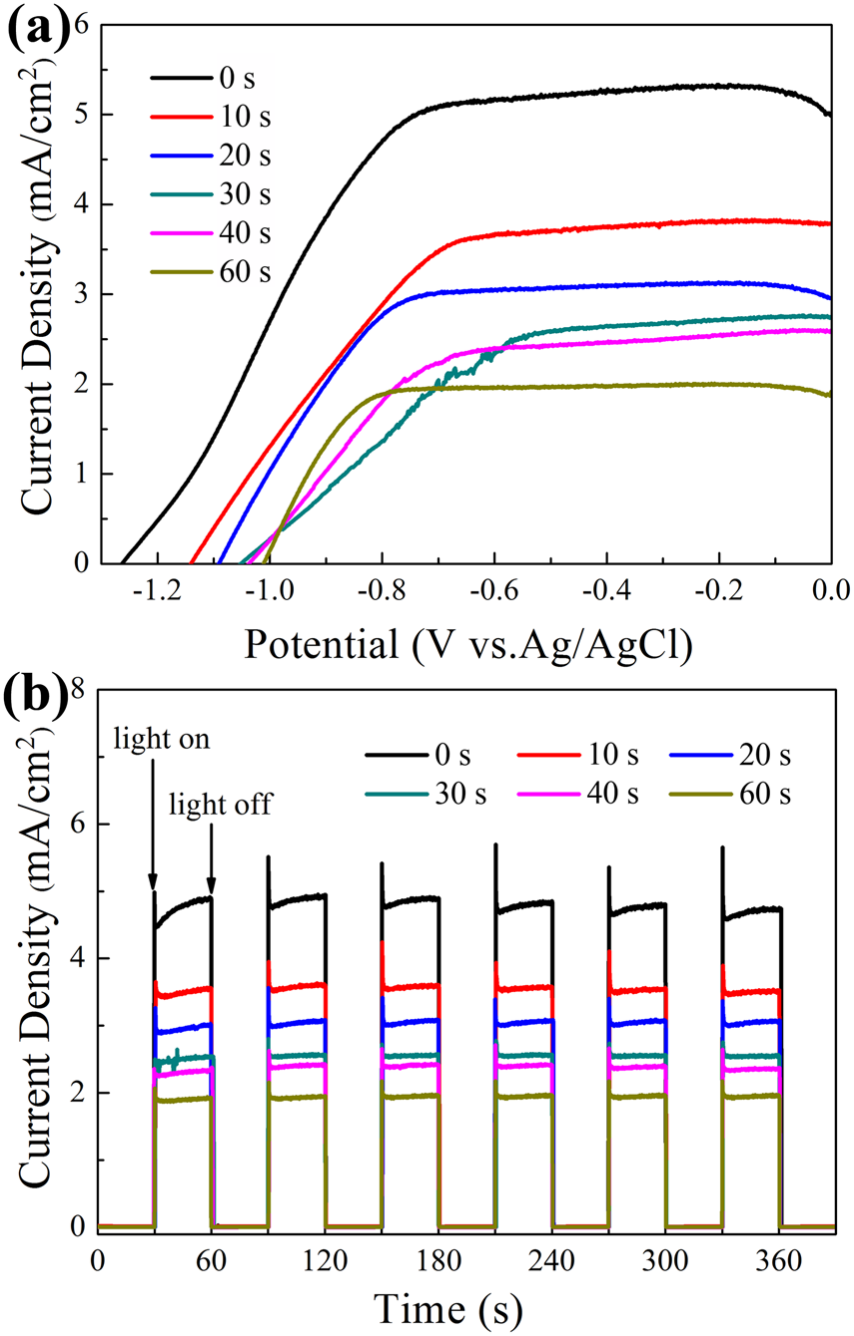
(**a**) Photocurrent density versus potential characteristics of the TiO_2_ NRAs/CdS/Au, (**b**) Photocurrent density versus time characteristics of the TiO_2_ NRAs/CdS/Au under 0 V versus Ag/AgCl bias.

*I*-*V* characteristics of TiO_2_ NRAs/CdS/Au are shown in Fig. 7(a). Photocurrent densities can be neglected for all samples without visible light irradiation. And the photocurrent density of TiO_2_ NRAs/CdS/Au decreases gradually with the increase of Au NPs under visible light irradiation. In addition, the open circuit potential (Voc) of the ternary TiO_2_ NRAs/CdS/Au becomes more positive compared with that of the binary TiO_2_ NRAs/CdS. More Au NPs deposited makes the Voc more positive. Figure 7(b) plots *I*-*T* characteristics of TiO_2_ NRAs/CdS/Au samples. All films exhibit a quick response to the on/off of the incident light. Under visible light irradiation, the photocurrent density descending trend for TiO_2_ NRAs/CdS/Au is in accordance with that in the *I-V* characteristics. It is strange that TiO_2_ NRAs/CdS/Au in our experiment displayed improved photocatalytic performance while deteriorative photoelectrochemcial property. According to previous reports, better photocatalytic performance is usually correlated with higher photocurrent density and more negative change of Voc^47–49^. To exclude the abnormal photoelectrochemcial property in our experiment was interfered by the difference in CdS among different samples, *I-V* curves of four TiO_2_ NRAs/CdS samples, which were prepared by the same method, were recorded in Fig. S4. It can be seen that the difference between the photocurrent densities is not significant. Error bars (inset in Fig. S4) were plotted by selecting statistics at −0.1 V, −0.2 V, −0.3 V, −0.4 V, −0.5 V, −0.6 V, −0.7 V and −0.8 V in the *I-V* characterization, whose errors are confined to about 0.2 mA/cm^2^. While as shown in Fig. 7(a), the photocurrent density difference between TiO_2_ NRAs/CdS and TiO_2_ NRAs/CdS decorated with different amounts of Au nanoparticles is obviously greater than 0.2 mA/cm^2^. It indicates that the abnormal phenomenon of the photocurrent density in Fig. 7 is not caused by the difference in CdS among different samples, rather, Au NPs should be responsible for it by extracting electrons from CdS. The following section will discuss the reason for the abnormal phenomenon in details.

**Figure 8:**
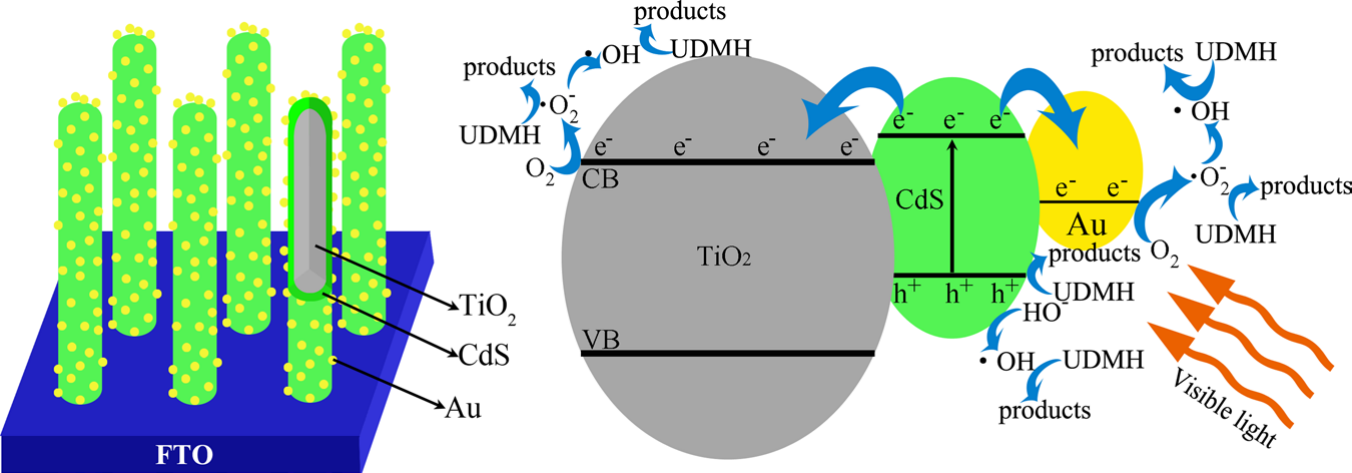
Schematic of carriers transfer and degradation process of UDMH for TiO_2_ NRAs/CdS/Au under visible light.

Based on the results in our experiment as well as previous literatures^43, 50–52^, the possible schematic of carriers transfer and degradation of UDMH process for TiO_2_ NRAs/CdS/Au is shown in Fig. 8. Under visible light irradiation, only CdS can be excited to generate electron and hole pairs. Since the Fermi energetic level of Au and the conduction band (CB) of TiO_2_ are both lower than the CB of CdS^38, 53^ as displayed in Fig. 8, electrons in the CB of CdS will transfer along two directions, *i.e*. to the Au NPs and to the CB of TiO_2_. And electrons in the CB of CdS seems to flow into Au NPs more easily, because the Fermi energetic level of Au is lower than the CB of TiO_2_^38, 53^. Meanwhile, a Schottky barrier at the interface of Au-CdS will be formed^39^, which can prevent the electrons flow back to CdS from Au NPs. Due to Au NPs extracting electrons from CdS, it should be noted that fewer electrons will be transferred to the CB of TiO_2_ in the ternary TiO_2_ NRAs/CdS/Au compared with that in the binary TiO_2_ NRAs/CdS. Therefore, fewer electrons will be transferred to the external circuit from TiO_2_, leading to the decreased photocurrent density in the ternary TiO_2_ NRAs/CdS/Au. It is reported that the electrons accumulated in the CB of TiO_2_ lead to the negative shift of the Fermi level^38^. If fewer electrons are accumulated in TiO_2_, we would expect a less negative shift of the Fermi level. So is the same with the Fermi level of TiO_2_ NRAs/CdS/Au, which will have a less negative shift compared with that of TiO_2_ NRAs/CdS. Accordingly, the Fermi level can be used to compare the Voc^38^, therefore, the Voc in the ternary TiO_2_ NRAs/CdS/Au will have a less negative shift than that in the binary TiO_2_ NRAs/CdS, *i.e*., the Voc will be more positive in the ternary TiO_2_ NRAs/CdS/Au than in the binary TiO_2_ NRAs/CdS. This abnormal photoelectrochemical results indicate that the TiO_2_-CdS-Au structure in our research is not helpful in energy output by converting solar energy.

Through aforementioned charge transfer process, the excited electron-hole pairs in CdS could be effectively separated. The electrons transferred to Au NPs and the CB of TiO_2_ could be trapped by dissolved oxygen molecules and then superoxide radical anions (·O_2_^−^) are generated^54^. ·O_2_^−^ could further react with electrons to form highly reactive hydroxyl radicals (·OH)^55^. The positive holes in the VB of CdS can oxide OH^−^ in the aqueous solution to yield ·OH^56^. ·O_2_^−^, ·OH and holes are all strong oxidizing free radicals, which can degrade UDMH into final products.

## Conclusion

In summary, we proposed a TiO_2_ NRAs/CdS/Au nanostructure that Au NPs, as the outermost layer, were loaded on the surface of TiO_2_ NRAs decorated by a thin layer of CdS. Due to the small particle size and the low distribution density, Au NPs could not cause obvious surface plasma resonance effect, therefore, the visible light absorption of TiO_2_ NRAs/CdS/Au was not improved. Rather, the deposited Au NPs working as electron sinks to extract electrons from CdS and accelerate the charge separation. As such, the ternary TiO_2_ NRAs/CdS/Au exhibits enhanced visible light photocatalytic ability. The best photocatalytic degradation rate of UDMH is obtained by the TiO_2_ NRAs/CdS/Au (30 s) sample, which is 1.4 times that by the binary TiO_2_ NRAs/CdS. The cycling stability of TiO_2_ NRAs/CdS is greatly improved after Au NPs decoration, whose photocatalytic capacity can maintain 95.86% after three cycles. An unexpected finding is that this structure is not conducive to the conversion of solar energy to electric energy owing to the Schottky barrier at the interface of the Au-CdS. This work may provide valuable preference in rational design of energy- and environment-related photocatalysts.

## Methods

### Sample preparation

Hydrothermal method was employed to grow vertically orientated TiO_2_ NRAs on FTO substrates (area 4.5 cm^2^). The detailed procedure is the same with our previous work^37^.

CdS NPs were successfully deposited on TiO_2_ nanorods through a Successive Ionic Layer Adsorption and Reaction (SILAR) method based on our previous work^31^. The TiO_2_ NRAs decorated by CdS NPs through 15 cycles of SILAR is chosen as the subject in this manuscript, which has been proved to achieve the best photocatalytic performance.

Au NPs were deposited on the surface of TiO_2_ NRAs/CdS films by ion sputtering (EMITECH, K550X). Through controlling the sputtering time, different amounts of Au NPs can be obtained.

### Characterization

X-ray diffraction (XRD, PANalytical) with Cu-Kα (λ = 0.15401 nm) radiation (40 kV, 40 mA) was collected to characterize the structure and crystallinity of the samples. The scanning speed of 5° min^−1^ was operated in a 2*θ* range of 20–80°. Raman spectra were measured to further clarify the composition of the samples using Ar^+^ (532 nm) laser excitation. Scanning electron microscopy (SEM) images were recorded on VEDAIIXMUINCN, and its energy dispersive X-ray spectroscopy (EDS) system was used to test the elementary composition. The microstructures of TiO_2_ NRAs/CdS/Au were studied by transmission electron microscopy (TEM) using a JEM-2100F transmission electron microscopy. The UV-vis absorption spectra were performed on a UV 1800 spectrophotometer (Shimadzu) with an FTO substrate as a blank. A Fluoromax-4 spectrophotometer was employed to record the photoluminescence (PL) spectra for samples with excitation wavelength at 350 nm.

Photocurrent curves were obtained under solar-simulated (AM 1.5 G filtered, 100 mW/cm^2^, CEL-HXF300) light source with a UV cutoff filter (providing visible light with λ ≥ 420 nm). A three-electrode configuration was used in a 250 mL quartz cell, including the prepared sample as the working electrode, a Pt foil as the counter electrode and a saturated Ag/AgCl as the reference electrode. Na_2_S aqueous solution (0.1 M) was used as the electrolyte. The working electrode was illuminated within an area of about 1.5 cm^2^ at zero bias voltage versus the Ag/AgCl electrode.

### Photocatalytic degradation of UDMH

Under visible light irradiation (λ ≥ 420 nm), the degradation of UDMH aqueous solution was carried out in an open reactor, which was placed in circulating cooling water with the temperature of 4 °C. The TiO_2_ NRAs/CdS/Au film with an area about 6 cm^2^ was used as the photocatalyst. During the photodegradation process, the concentration of UDMH left in the aqueous was measured by spectrophotometry every 30 min. Before measurement, UDMH should first react with amino ferrocyanide sodium in a weakly acidic aqueous solution, and a red complex was formed with its maximum absorbance at 500 nm. The color depth of the red complex is proportional to the content of UDMH. Therefore, the concentration of UDMH left in the aqueous can be obtained by measuring the red complex. Detailed procedures for the photocatalytic degradation and the measurement of UDMH have been reported in our previous study^31^.

## Electronic supplementary material

SUPPLEMENTARY INFO
