# Supplementary material for: Enhanced visible light photocatalytic performance of CdS sensitized TiO2 nanorod arrays decorated with Au nanoparticles as electron sinks
Source: Sci Rep. 2017 Apr 20;7:973. doi: 10.1038/s41598-017-01124-5 (PMC5430509; doi:10.1038/s41598-017-01124-5)
Supplement: Supplementary file 1 — SUPPLEMENTARY INFO [file 41598_2017_1124_MOESM1_ESM.doc]

*Supporting Information*

**Enhanced visible light photocatalytic performance of CdS sensitized TiO_2_ nanorod arrays decorated with Au nanoparticles as electron sinks**

Xin Gao^1^, Xiangxuan Liu^1*^, Zuoming Zhu^2^, Ying Gao^2^, Qingbo Wang^2^, Fei Zhu^3^ and Zheng Xie^1,3*^

1. High-Tech Institute of Xi’an, Xi’an, 710025, China.

2. High-Tech Institute of Beijing, Beijing, 100085, China.

3. State Key Laboratory of New Ceramics and Fine Processing, School of Materials Science and Engineering, Tsinghua University, Beijing, 100084, China.

*Correspondence should be addressed to Xiangxuan Liu and Zheng Xie: [liuyc1214@sina.com](mailto:liuyc1214@sina.com) and [xiezheng10@tsinghua.org.cn](mailto:xiezheng10@tsinghua.org.cn)


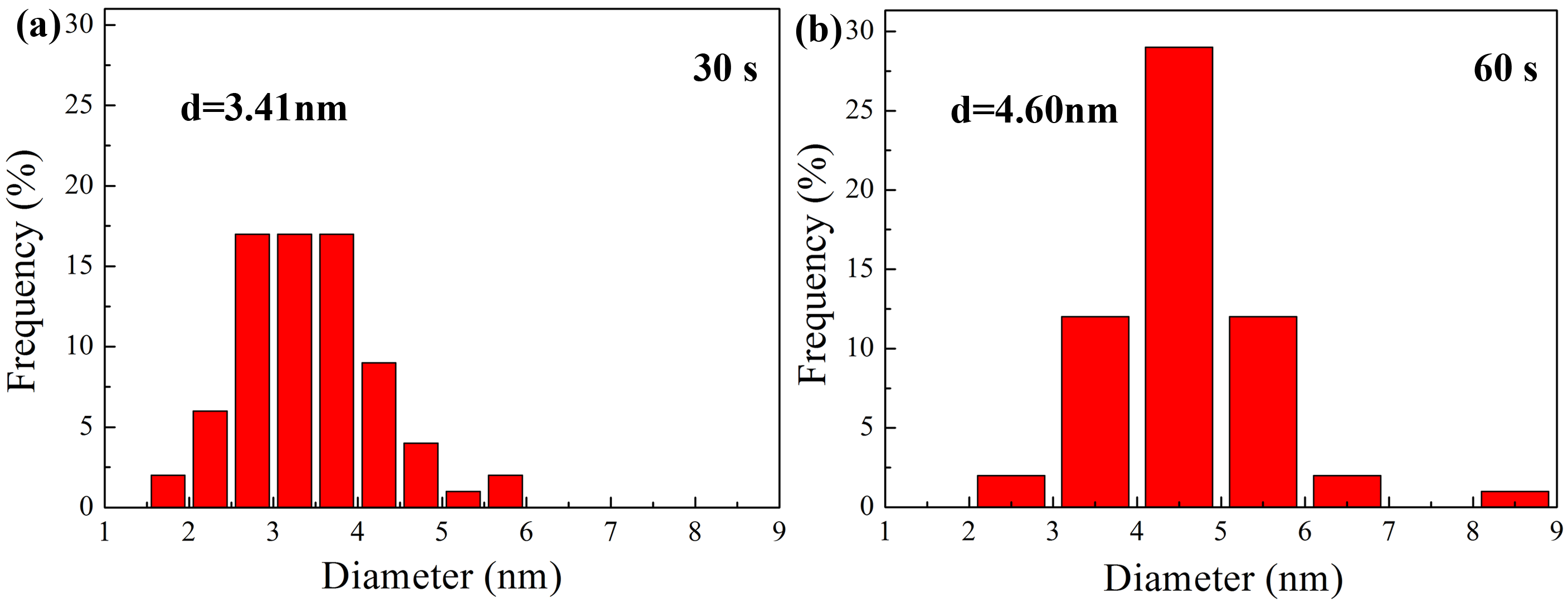


Figure S1. Size distribution of Au NPs in TiO_2_ NRAs/CdS/Au:

(a) 30 s and (b) 60 s


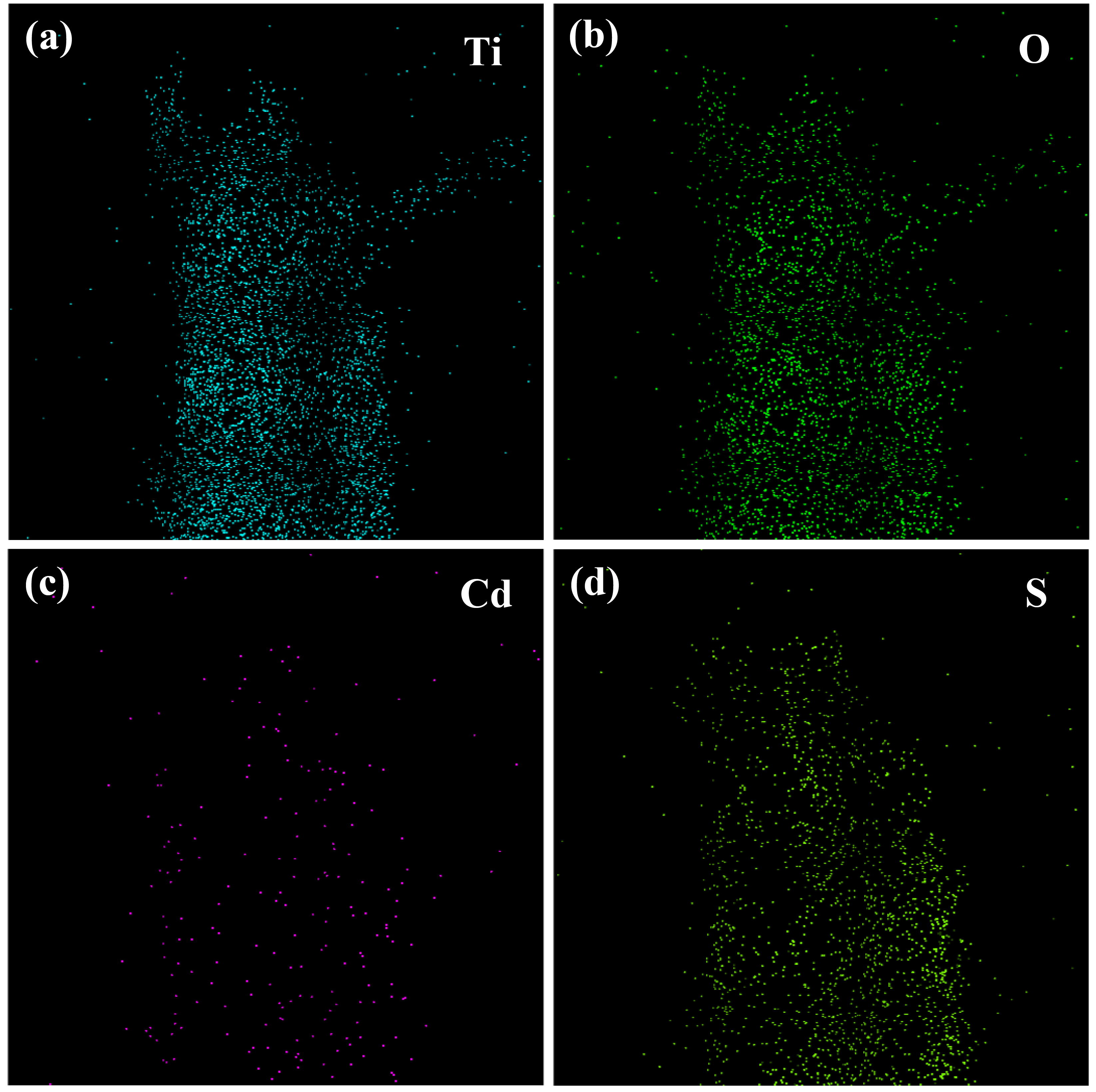


Figure S2. TEM mapping of TiO_2_ NRAs/CdS/Au(60 s): (a) Ti, (b) O, (c) Cd and

(d) S elements


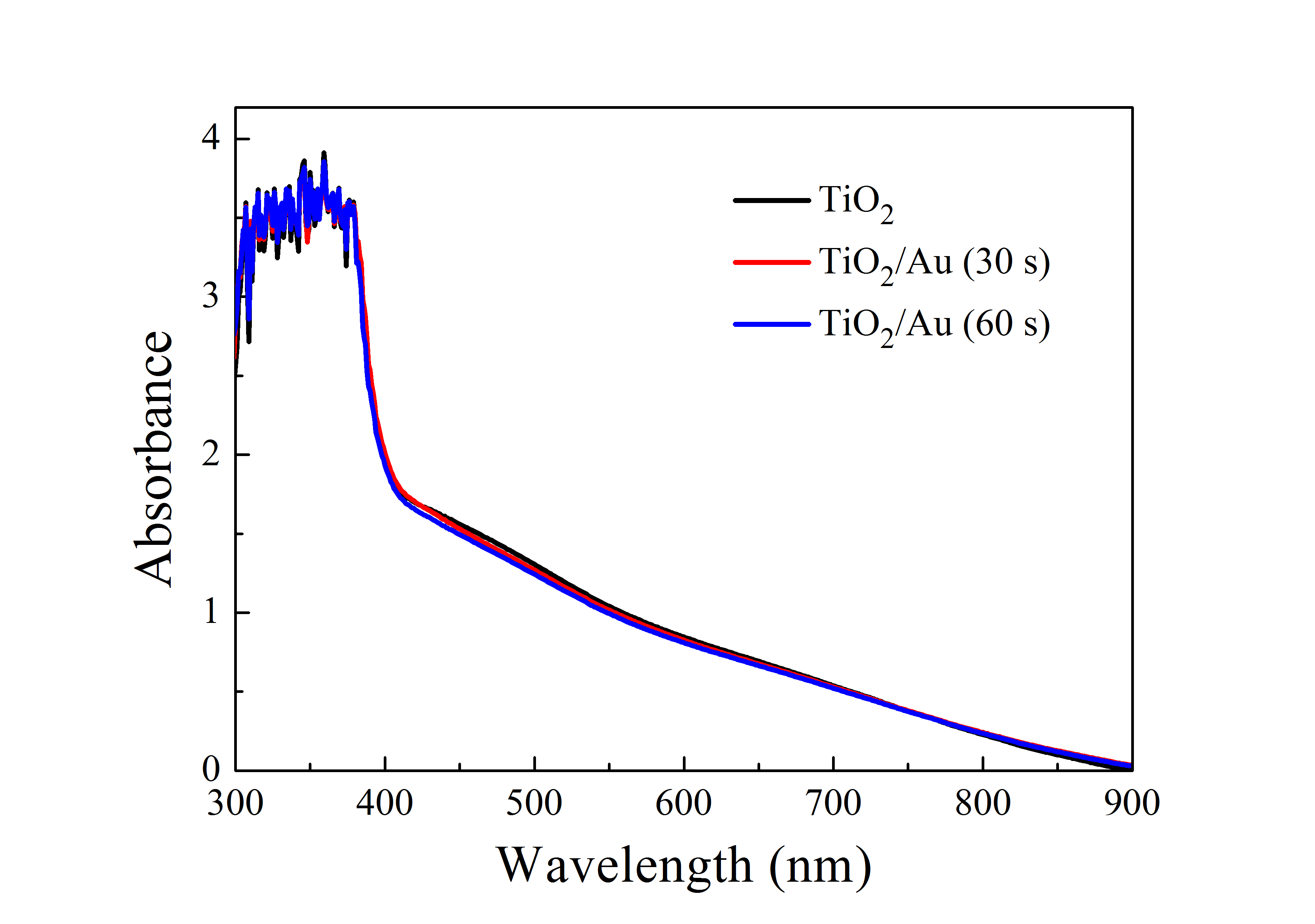


Figure S3. UV-vis absorption spectra of TiO_2_ NRAs and TiO_2_ NRAs/Au


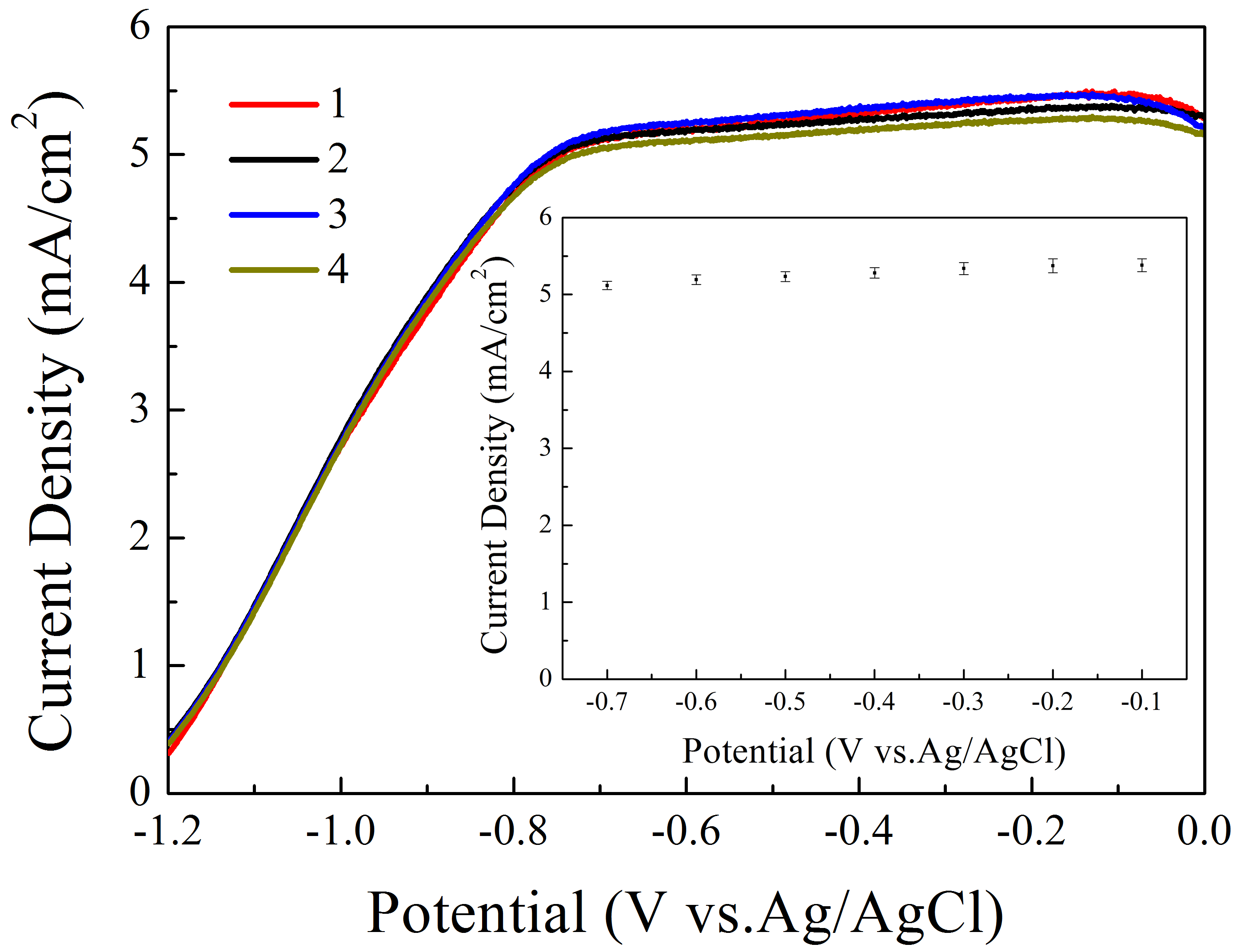


Figure S4. Photocurrent density versus potential characteristics of different TiO_2_ NRAs/CdS samples, inset is the error bar
